# Wash for Chapped Hands

**Published:** 1892-12

**Authors:** 


### Wash for Chapped Hands.

Wendell recommends the following wash for chapped hands:

| | |
|---|---|
| ℞ Green soap, . . . | 1 part. |
| Compound benzoin tincture, | 4 parts. |
| Glycerine, . . . . | 8 parts. |
| Rose water, . . . . | 16 parts. |

